# Bacterial and viral etiology of childhood diarrhea in Ouagadougou, Burkina Faso

**DOI:** 10.1186/1471-2431-13-36

**Published:** 2013-03-19

**Authors:** Isidore Juste O Bonkoungou, Kaisa Haukka, Monica Österblad, Antti J Hakanen, Alfred S Traoré, Nicolas Barro, Anja Siitonen

**Affiliations:** 1 Bacteriology Unit, National Institute for Health and Welfare (THL), P.O. Box 30, Helsinki 00271, Finland; 2 Laboratoire de Biologie Moléculaire, d’Epidémiologie et Surveillance des Bactéries et Virus transmis par les Aliments, CRSBAN/UFR-SVT, Université de Ouagadougou, Ouagadougou 03 BP 7021, Burkina Faso; 3 Laboratoire National de Santé Publique, Ouagadougou 09 BP 24, Burkina Faso; 4 Department of Food and Environmental Sciences, Division of Microbiology, University of Helsinki, P.O. Box 56, Helsinki FI-00014, Finland; 5 Antimicrobial Resistance Unit, National Institute for Health and Welfare (THL), P.O. Box 57, Turku 20521, Finland

## Abstract

**Background:**

Diarrhea is the most frequent health problem among children in developing countries. This study investigated the bacterial and viral etiology and related clinical and epidemiological factors in children with acute diarrhea in Ouagadougou, Burkina Faso.

**Methods:**

Stool specimens were collected from 283 children under 5 years of age visiting hospital due to acute diarrhea and from 60 healthy controls of similar age. Pathogens were investigated by using conventional culture techniques, PCR and immunochromatographic testing. *Salmonella* and *Shigella* strains were serotyped and their susceptibility to 23 antimicrobial agents was determined by the agar dilution method.

**Results:**

At least one pathogen was detected in 64% of the 283 patients and in 8% of the 60 controls (*p* < 0.001). Rotavirus was found in 30% of the patients, followed by diarrheagenic *Escherichia coli* (24%), *Salmonella enterica* ssp. *enterica* (9%), *Shigella* spp. (6%), adenovirus (5%) and *Campylobacter* spp. (2%). Multiple pathogens were found in 11% of the patients and in 2% of the controls (*p =* 0.028). Viruses were found mainly in children of ≤ 2 years of age, whereas bacteria were equally prevalent among all the age groups. Viral infections occurred mostly during the cool dry season and the bacterial infections during the rainy season. Fever (64%) and vomiting (61%) were the most common symptoms associated with diarrhea. Only one *Salmonella* strain was resistant to nalidixic acid and ciprofloxacin. Of the *Shigella* strains, one was resistant to nalidixic acid but 81% to trimethoprim- sulfamethoxazole, 63% to streptomycin and 50% to ampicillin. Most of all the other *Salmonella* and *Shigella* strains were sensitive to all antimicrobials tested.

**Conclusion:**

Rotaviruses and diarrheal *E. coli* were the most predominant pathogens associated with acute diarrhea in Burkinabe children. Constant antimicrobial surveillance is warranted to observe for the emergence of enteric bacteria resistant to antimicrobials that are important in treatment also of severe infections.

## Background

Acute diarrheal disease is a major public health problem throughout the world, with over two million deaths occurring each year, and affecting mostly children under 5 years of age in developing countries [1,2]. These diseases are especially common in developing countries with poor hygiene and sanitation and with limited access to safe drinking water. Underlying conditions, such as malnutrition, which increase the risk of contracting diarrhea, are also common in these countries. These factors may result in a significant disease burden and economic effect due to direct medical costs, loss of work, lower quality of life and mortality. The etiological agents for acute diarrhea include a wide range of viruses, bacteria and parasites. In Burkina Faso, diarrhea is the third most common cause of young children to visit a health care centre, but knowledge of its causative agents is limited. In our previous reports, rotavirus and diarrheagenic *Escherichia coli* (DEC) were found to be common causes of childhood diarrhea [3,4]. Recently, two studies using conventional diagnostic techniques were conducted in Ouagadougou to determine the occurrence of certain viruses, bacteria and parasites in children with diarrhea [5,6]. Yet, a comprehensive study on the actual significance of the etiological agents in acute diarrhea that also includes a control group of healthy individuals has not been performed. Here, we report on a one-year follow-up study using a combination of conventional and molecular methods to search for *Salmonella* spp., *Shigella* spp., *Campylobacter* spp., *Yersinia* spp. and the most common pathogroups of DEC including enteropathogenic *E. coli* (EPEC), enterotoxigenic *E. coli* (ETEC), Shiga toxin-producing *E. coli* (STEC), enteroinvasive *E. coli* (EIEC) and enteroaggregative *E. coli* (EAEC) as well as rotavirus and adenovirus in stool samples of children with and without diarrhea in urban Burkina Faso. In addition, seasonality of diarrhea, demographics and clinical features related to the pathogen-associated diarrhea were investigated. We also determined the serotypes and antimicrobial resistance patterns of the *Salmonella* and *Shigella* findings.

## Methods

### Study design and sampling

The study was conducted at Centre Médical avec Antenne Chirugicale (CMA) du Secteur 30 in the capital city of Ouagadougou, Burkina Faso. CMA du secteur 30, located in the Bogodogo district is one of the four secondary health care centers in Ouagadougou and its pediatric ward has a capacity of 30 beds and admits some 2300 children each year. The facility provides primary health care for 548 000 persons, with 81 000 (15%) being children under 5 years of age (http://www.sante.gov.bf). Most of the patients who visit this facility are poor and come from peripheral areas of the city. Burkina Faso has a tropical climate with two very distinct seasons: a rainy season of approximately four months (May/June to September) and a dry season of eight months (October to April/May with a cooler period from December to February).

Between January 2009 and January 2010, 283 children aged under 5 years with acute diarrhea were enrolled at the CMA of sector 30. Diarrhea was defined as three or more loose, liquid, or watery stools or at least one bloody loose stool passed in 24 hours [7]. The control subjects, randomly selected during the same time period, were 60 children with no history of diarrhea for at least 21 days prior to visiting the same health centre for reasons other than diarrhea. Demographic information (age and sex) and clinical symptoms (fever, vomiting and dehydration) were recorded for each child using a questionnaire. All children were clinically evaluated by general practitioners following a local adaptation of the World Health Organization (WHO) strategy for the management of diarrhea. Dehydration (severe, some, no dehydration) was classified according to the WHO guidelines. Stool samples were collected by trained healthcare personnel using sterile stool containers and transferred to the Microbiology Laboratory at the National Public Health Laboratory, Ouagadougou, on ice packs and were processed within 4 h of collection.

### Pathogen detection, isolation and identification

Stool samples were cultured on Hecktoen Enteric agar for *Shigella* and *Salmonella* strains, modified charcoal cefoperazone deoxycholate agar (mCCDA) for *Campylobacter* spp. and cefsulodin-irgasan-novobiocin (CIN) agar for *Yersinia* spp. Bacterial isolates were identified according to the standard microbiological procedures [8]. *Shigella* and *Salmonella* strains were subsequently sent to the National Institute for Health and Welfare (THL), Finland, for serotyping and antimicrobial susceptibility testing. For detection of diarrheagenic *E. coli* (DEC)*,* the samples were cultured on Sorbitol MacConkey agar (SMAC), and DEC were investigated using multiplex PCR for 16 genes present in *E. coli* in general and in EPEC, ETEC, EIEC, STEC or EAEC specifically in a single PCR reaction, as described previously [4]. Detection of viruses (group A rotavirus and adenovirus serotypes 40/41) was carried out using a one-step rotavirus and adenovirus test of human feces (SD Bioline Rota/Adeno®; Standard diagnostics, Inc., Kyonggi-Do, South Korea).

### Antimicrobial susceptibility testing of the bacterial isolates

Antimicrobial susceptibility of *Salmonella* and *Shigella* isolates were tested by agar dilution technique with 23 antimicrobials (ampicillin, nalidixic acid, ciprofloxacin, cefuroxime, cefotaxime, cefotaxime-clavulanic acid, cefepime, cefepime-clavulanic acid, cefoxitin, piperacillin-tazobactam, aztreonam, meropenem, ertapenem, trimethoprim-sulfamethoxazole 1:19, tetracycline, tigecycline, streptomycin, gentamicin, tobramycin, azithromycin, nitrofurantoin, colistin, chloramphenicol). EUCAST breakpoints v.1.3 [http://www.eucast.org/] were used where available; if not available, EUCAST ECOFF values, or as the last choice, the CLSI breakpoint [9] (for tetracycline) were used. Most antimicrobials were from Sigma (St. Louis, Missouri, USA), except ciprofloxacin from Fluka (Buchs, Swizerland) and cefepime from Bristol-Myers Squibb (Syracuse, N.Y., USA). Ertapenem (MerckSharp&Dohme Ltd. Hertfordshire, UK), meropenem (Astra Zeneca, Espoo, Finland) and tigecycline (Wyeth Pharmaceuticals, Hampshire, UK) were available only as clinical preparations. They contained sodium, sodium carbonate anhydrate, and lactose monohydrate, respectively, in addition to the active substance; this was taken into consideration when preparing the stock solutions. Mueller-Hinton II agar (Becton Dickinson BBL, Le Pont de Claix, France) was used for all tests. Control strains were *E. coli* ATCC 25922, *E. coli* ATCC 35218, *Pseudomonas aeruginosa* ATCC 27853, *Enterococcus faecalis* ATCC 29212, and *Staphylococcus aureus* ATCC 29213.

### Ethical considerations

The study protocol was approved by the Ethical Committees of Burkina Faso and the Hospital District of Helsinki and Uusimaa, Finland. An informed verbal consent was obtained from the parents/guardians of every child before taking the stool samples.

### Statistical analysis

The χ^2^ test or Fisher’s exact test of OpenEpi version 2.3.1 was used to determine the statistical significance of the data. A P value of <0.05 was considered statistically significant.

## Results

### Demographics

Of the 283 children with diarrhea, 49% were aged 0–12 months, 34% were aged 13–24 months and 17% were aged 25–59 months old. In the control group with 60 children, there were significantly (*p* < 0.001) more older children than in the patient group, the corresponding percentages being 33%, 22% and 45%, respectively (Table 1). Of the patients 53% and of the controls 48% were males.

**Table 1:** Distribution of the diarrheal and control children by age groups

**Age group (months)**	**No. (%) of children**		**Total **^**a **^**(n = 343)**
	**Diarrhea**	**Control**	
	**(n = 283)**	**(n = 60)**	
0-12	138 (49)	20(33)	158(46)
13-24	96 (34)	13(22)	109(32)
25-59	49(17)	27(45)	76 (22)

^**a**^*p* <0.001, numbers of diarrheal children and control children were not equal in each age group.

### Occurrence of enteric pathogens

At least one pathogen was detected in 64% of the 283 diarrheal children and in 8% of the 60 control children (*p* < 0.001; Table 2). Due to the simultaneous occurrence of two, three or four pathogens in stool samples of some patients, a total of 218 pathogens were detected in 181 patients and 6 pathogens in 5 controls.

**Table 2:** Occurrence of the enteropathogens in the stool samples of 283 children with diarrhea (cases) and of 60 children without diarrhea (controls) in Ouagadougou, Burkina Faso

	** No. (%) of cases and controls**		
**Microbes**	**Cases n = 283**	**Controls n = 60**	**p-value**
**Children with any pathogens**	181(64)	5(8)	p < 0.001
**Bacteria**	112(40)	5(8)	p < 0.001
Diarrheagenic *E. coli*	67(24)	4(7)	p = 0.005
EAEC	34(12)	4(7)	NS
EPEC	22(8)	0(0)	p = 0.025
ETEC	10(4)	0(0)	NS
EIEC	1(0.4)	0(0)	NS
STEC	1(0.4)	0(0)	NS
*Salmonella* spp	24(9)	1(2)	NS
*Shigella* spp	16(6)	0(0)	NS
*Campylobacter* spp	5(2)	0(0)	NS
**Virus**	99(35)	1(2)	p < 0.001
Rotavirus	85(30)	1(2)	p < 0.001
Adenovirus	14(5)	0(0)	NS
**Co-infections**	30(11)	1(2)	p = 0.028
Bacteria-Bacteria	17(6)	0(0)	NS
Virus-Virus	3(1)	0(0)	NS
Bacteria-Virus	10(4)	1(2)	NS

Significant differences in the distribution between the two groups is indicated with the p-values. *NS* non-significant.

Of the patients, 40% had bacterial etiology compared with 8% of the controls (*p* < 0.001). Genes indicating *E. coli* pathogroups were found in 24% of the patients *vs*. 7% of the controls (*p* = 0.005), followed by strains of various *Salmonella enterica* ssp. *enterica* serotypes (9% *vs*. 2%), *Shigella* spp. (6% *vs*. 0%) and *Campylobacter* spp. (2% *vs*. 0%). No *Yersinia* spp. were found.

Of the *E. coli* pathogroups detected, the most frequent was EAEC (12% in patients *vs*. 7% in controls), followed by EPEC (8% *vs*. 0%; *p* = 0.025) and ETEC (4% *vs*. 0%). STEC and EIEC were found only in one patient each.

The 25 *Salmonella* isolates represented 15 different serotypes: *S.* Typhimurium (in 6 patients), *S.* Cubana (4), *S.* Muenster (3), and *S.* Angers, *S.* Banana, *S.* Dublin, *S.* Kentucky, *S*. Montevideo *S.* Ouakam, *S.* Soerenga*, S.* Poona, S*.* Stanley *S.* Tamberma, *S.* Vilvoorde, and *S*. Typhi (in 1 patient each).

Of the 16 *Shigella* isolates, nine were *S. flexneri* (6 cases of *S. flexneri* 2a and 1 case of *S. flexneri* 3a*, S. flexneri* 1b and *S. flexneri* 1c), five were *S. boydii* (4 cases of *S. boydii* 18 and 1 case of *S. boydii* 4) and two were *S. sonnei* strains. Of the five *Campylobacter* isolates, three were *C. jejuni* and two were *C. coli*.

Viral etiology was found in 35% of the patients and in 2% of the controls (*p* < 0.001; Table 2) with rotavirus being the most common finding in both groups (30% *vs*. 2%; *p* < 0.001). Adenovirus serotype 40/41 was found only in patients (5%). Multiple (bacterial and/or viral) pathogens were detected in 11% of the patients and in 2% of the controls (*p* = 0.028). Of the 30 patients who were co-infected, 25 had two pathogens, three patients had three and two patients had four pathogens.

Bacterial and viral enteropathogens were detected in all age groups of the patients, but most commonly (76%) they occurred in the group aged 0–24 months (Table 3). The most prevalent pathogens, rotavirus and DEC, were detected as sole pathogens mainly in the age group of 0–12 months and *Salmonella* as well as *Shigella* in children older than 12 months (Table 3). In the control group, all pathogens were detected in the children older than 36 months (data not shown).

**Table 3:** Age groups and clinical characteristics by pathogens of 181 diarrheal children with a single pathogen (151 children) or several (30 children) enteric pathogens

		**Characteristics**							
**Microbes**	**Number of cases**	**Age in months**			**Diarrhea type**			**Symptoms**	
		**0-12**	**13-24**	**25-59**	**Watery**	**Bloody**	^**a**^**Fever**	**Vomiting**	**Dehydration**
**Bacteria**	78	27(35)	27(32)	24(33)	75(96)	3(4)	46(59)	46(59)	34(43)
DEC^b^	41	21(51)	12(29)	8(20)	40(98)	1(2)	17(41)	20(49)	10(24)
Salmonella spp.	20	3(15)	8(40)	9(45)	20(100)	0(0)	15(75)	14(70)	13(65)
Shigella spp.	14	3(21)	6(43)	5(36)	13(97)	1(7)	13(87)	9(60)	9(60)
Campylobacter spp.	3	0(0)	1(33)	2(67)	2(67)	1(33)	2(50)	3(75)	2(67)
**Virus**	73	44(60)	22(30)	7(10)	73(100)	0(0)	50(68)	44(60)	24(37)
Rotavirus	65	42(65)	20(31)	3(5)	65(100)	0(0)	46(71)	40(62)	22(34)
Adenovirus	8	2(25)	2(25)	4(50)	8(100)	0(0)	4(50)	4(50)	2(25)
**Co-infection**	30	7(23)	10(33)	13(43)	28(93)	2(7)	20(67)	20(63)	18(60)
**Total**	181	78(43)	59(33)	44(24)	176(97)	5(3)	116(64)	110(61)	76(42)

^a^Fever if the temperature of the body rises to ≥38°C.

^b^DEC Diarrheagenic *Escherichia coli.*

### Clinical characteristics and seasonality

Of the diarrheal children with enteropathogens, 97% had watery and 3% had bloody diarrhea (Table 3). Fever occurred in 64% of the diarrheal children, most commonly in children with shigellosis, salmonellosis or rotaviral infection. Vomiting was reported for 61% and dehydration for 42% of the diarrheal children.

**Figure 1:**
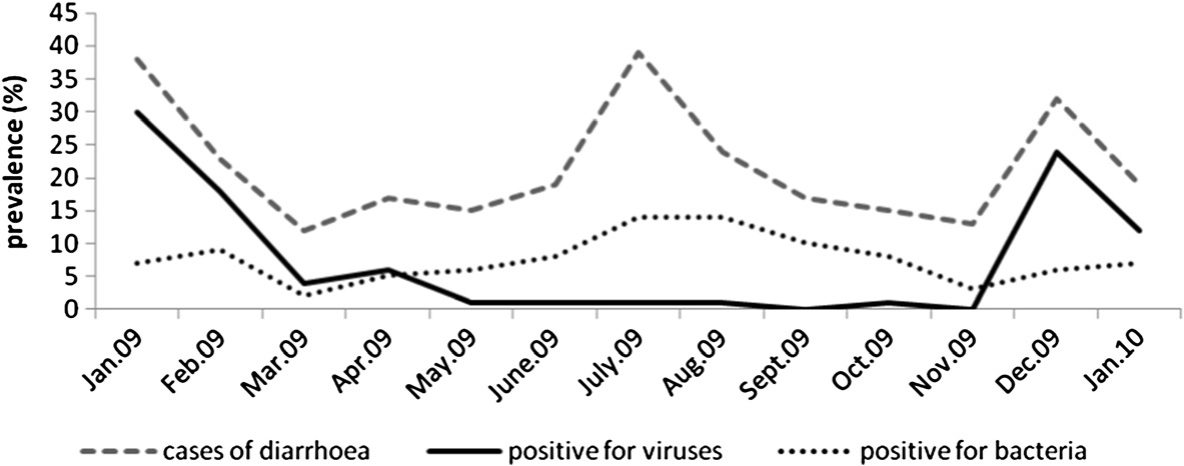
Seasonality of the acute diarrhea and the detection frequency of enteropathogenic bacteria and viruses in the stool samples of 283 children suffering from gastroenteritis in Ouagadougou, Burkina Faso.

The elevated incidence of diarrhea correlated with the occurrence of the cool dry season (December to February) and the warm rainy season (June to August) (Figure 1). The viral infections occurred mainly during the dry season and bacterial infections during the rainy season.

### Antimicrobial resistance

In 25 *Salmonella* and 16 *Shigella* strains, resistance was detected to chloramphenicol in 5 (20%) *Salmonella* and 4 (25%) *Shigella* strains, to ampicillin in 7 (28%) *Salmonella* and 8 (50%) *Shigella* strains, to streptomycin in 6 (24%) *Salmonella* and 10 (63%) *Shigella* strains and to trimethoprim-sulfamethoxazole in 5 (20%) *Salmonella* and 13 (81%) *Shigella* strains. One *Salmonella* serotype Kentucky strain was resistant to nalidixic acid (MIC > 128) and ciprofloxacin (MIC = 8), and one *S. boydii* serotype 4 strain was resistant to nalidixic acid (MIC >128).

## Discussion

We used a combination of conventional and molecular diagnostic techniques to investigate the occurrence of bacterial and viral enteropathogens in stool samples of Burkinabe children with and without diarrhea living in an urban area.

The prevalence of diarrheal pathogens was significantly higher in patients (64%) than in control children (8%). Similar results have also been reported from other countries [10,11], showing the importance of bacterial and viral infections as a cause of childhood diarrhea. In Ouagadougou, the prevalence of pathogens was higher in patients than was detected in the previous studies carried out in Burkina Faso [5,6]. This is probably because, unlike the other reports, we investigated also the presence of different DEC pathogroups and *Campylobacter*, and these findings increased the frequency of detecting the causative agents of diarrhea. Rotavirus was the most frequently detected enteropathogen, supporting the well-documented role of rotavirus in childhood diarrheal disease in Burkina Faso as in some other developing countries [3,5,12-14]. A majority (90%) of the rotavirus infections occurred among children under 2 years of age. The occurrence of the other virus investigated in this study, adenovirus, was relatively low (5%) but somewhat higher than the 2% found in the previous study in Burkina Faso [6]. A comparable prevalence of 5% has been reported from both Tanzania and Tunisia [15,16].

The various DECs investigated by multiplex PCR were the second most common group of enteropathogens detected in our study. Their prevalence (24%) was slightly lower than that (31%) found in our previous study in children living in the same urban area [4]. EAEC was the most commonly detected *E. coli* pathogroup in both patients and control children, suggesting that EAEC is actually not associated with diarrhea. Also other studies [17,18] have reported similar results, whereas some studies have concluded that EAEC are causes of diarrhea [10,11]. Our results together with the results from previous studies conducted in Nigeria and Ghana suggest that EAEC is endemic in West Africa [4,19,20], but is not a primary cause of diarrhea. The frequencies of EPEC (8%) and ETEC (4%) were lower than in our previous report (16% and 13%, respectively) [4]. However, that study was conducted during the rainy period, which may explain the differences. Nevertheless, these *E. coli* pathogroups can be considered as major bacterial causes of infantile diarrhea in developing countries [19,21,22].

In the present study, *Salmonella enterica* ssp. *enterica* comprising several serotypes was isolated from 9% of the patients. This percentage is high compared to the previous studies carried out in Burkina Faso (2%) [5] and in southeastern and eastern Africa, e.g. in Mozambique and Tanzania in which the prevalence was approximately 3% [23,24]. *Shigella* spp. were isolated from 6% of the diarrheal children with a predominance for *S. flexneri*. Other researchers in Burkina Faso [5], in Tanzania [24] and in Jordan [25] reported similar rates for shigellosis. Our study was the first one to detect *Campylobacter jejuni*/*coli* in Burkina Faso; this pathogen is not searched for in the routine laboratory investigations for etiology of diarrhea. Our results show that *Salmonella*, *Shigella* and *Campylobacter* occur nearly exclusively in symptomatic children. A larger sample size is required however to confirm the statistical significance of this result.

Co-infections with two or more enteropathogens were common, making it more difficult to define the exact etiology of diarrhea and the exact cause behind symptomatic children. Moreover, parasites and other viral enteropathogens, such as norovirus and sapovirus, which were not included in our study, can cause enteric infections [14,26] and may be responsible for illness in some of the 36% of cases with no etiologic diagnosis. In our study, the children in the control group were older than the children in the patient group. However, 76% of all pathogens were found in the patients aged 0–24 months, although all the pathogens in the control group were found in older children. This suggests that young children are susceptible to the enteropathogens, but with increasing age and living in the endemic area, they acquire some degree of resistance to enteric infections. When the association of the enteropathogens with the age of diarrheal children and their clinical features were examined, the most prevalent pathogens rotavirus and DEC were most common as sole pathogens in the youngest age group (0–12 months old children), whereas *Salmonella* and *Shigella* were mainly found as sole pathogens in children older than one year, as also reported by Nguyen et al. [27]. The occurrence of watery diarrhea was far more common than bloody diarrhea. Also, other symptoms were, in general, similar regardless of the detected pathogen, as has been reported before [25].

The antimicrobial susceptibility tests showed that only 20% of the *Salmonella* strains but 81% of the *Shigella* strains were resistant to trimethoprim-sulfamethoxazole, which is the most commonly used antimicrobial treatment for the Burkinabe children already previous to their enrollment in to the health center [5]. However, the resistance rates to the newer generation antimicrobials as detected in our study were still very low.

## Conclusion

In conclusion, this study highlights the importance of the bacteria and viruses as the cause of diarrhea diseases in young children in Burkina Faso. Rotaviruses and *E. coli* pathogroups were the most common etiological agents in acute diarrhea during the one year study period. Seasonal variation was seen in the occurrence of the etiological agents. Constant antimicrobial surveillance is warranted to observe the potential emergence of the enteric bacteria resistant to the antimicrobials that are presently important in treatment of severe infections.

## Competing interests

The authors declare that they have no competing interests.

## Authors’ contributions

IJOB was responsible for initiation of the study, for collection of the specimens and clinical information as well as for data analysis. Laboratory investigations were performed by IJOB and MÖ under the guidance of KH, AJH and AS. NB, AST, KH and AS participated in development of the research proposal, data analysis and preparation of the manuscript. All the authors have read and approved the final manuscript

## Pre-publication history

The pre-publication history for this paper can be accessed here:

http://www.biomedcentral.com/1471-2431/13/36/prepub
